# Does measurement of exercise/rest calf muscle perfusion reserve with first-pass contrast-enhanced MRI in peripheral arterial disease perform better than exercise-only perfusion ?

**DOI:** 10.1186/1532-429X-13-S1-P385

**Published:** 2011-02-02

**Authors:** Ronny S Jiji, Amy M West, Frederick H Epstein, Patrick F Antkowiak, Craig H Meyer, Arthur Weltman, Joseph M DiMaria, Jennifer R Hunter, John M Christopher, Christopher M Kramer

**Affiliations:** 1 University of Virginia, Charlottesville, VA, USA

## Background

Perfusion index (PI) measured by contrast-enhanced exercise calf perfusion MRI distinguishes normal subjects from those with PAD. Rest perfusion can be measured, allowing calculation of perfusion reserve (PR) and perfusion index reserve (PIR).

## Objective

To determine whether perfusion or perfusion reserve with first-pass contrast enhanced MRI is the most reproducible measure of exercise calf blood flow.

## Methods

Sixteen healthy subjects and 3 symptomatic PAD patients (ABI 0.4-0.9) underwent contrast-enhanced perfusion MRI of the calf before and after plantar-flexion exercise using an MR-compatible pedal at 50 rpm for up to 20 minutes or until limiting symptoms. Contrast-enhanced first-pass images were obtained on a 3T Siemens Trio scanner by infusion of 0.1mM/kg of gadolinium-DTPA followed by a 20 mL saline flush at 4 mL/second. A spoiled gradient echo dual contrast sequence with slices positioned 32 mm apart allowed for simultaneous acquisition of arterial input and muscle perfusion images. Time-intensity curves (TIC) were generated using ARGUS software (Siemens) for arterial input and the muscle group with the highest signal intensity (tissue function, TF). PI was measured as ratio of the slopes of the TF TIC/arterial input TIC. PR was defined as exercise TF/resting TF. PIR was calculated as exercise PI/rest PI. Ten of the controls and all 3 patients underwent repeat studies on a separate day. For 7 controls and all patients, measurements were normalized to proton density.

## Results

Participant mean age was 60±8. ABI in PAD was 0.70±0.11. Exercise time was 816±453 seconds for normals and 567±516 seconds for PAD. The anterior tibialis commonly demonstrated the highest signal intensity. Median exercise PI and rest PI were 0.29 (25^th^, 75^th^ percentiles = 0.22, 0.52) and 0.018 (0.009, 0.052) in 16 healthy subjects. Mean PR and PIR for normal subjects were 30±20 and 21±17, respectively.

**Figure 1:**
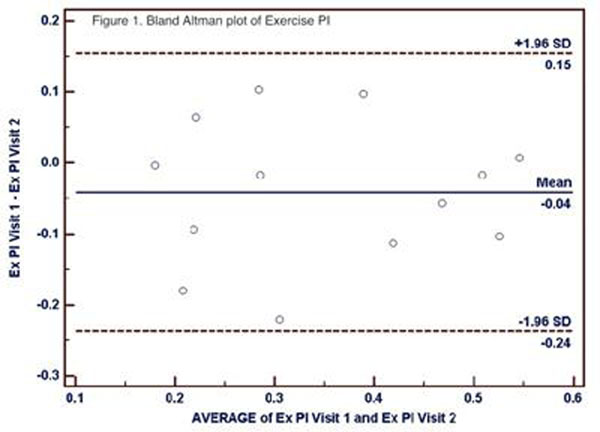
land Altman plot of Exercise PI

For normal and PAD patients with repeat studies, intraclass correlation (ICC) for exercise PI was similar for both normalized and non-normalized measurements (Table 1). See Figure 1 for Bland Altman limits of agreement for exercise PI. The ICC for rest PI improved with normalization (0.64 vs 0.77). The ICC for PR and PIR were weaker than for PI.

**Table 1:** Intraclass Correlation Coefficienct

	ICC
Exercise PI (n=13)	0.73
Exercise PI norm (n=10)	0.73
Rest PI (n=13)	0.64
Rest PI norm (n=10)	0.77
Perfusion Reserve (n=13)	0.54
Perfusion Index Reserve (n=13)	0.22
*norm=normalized to proton density	

## Conclusions

Exercise PI remains the most reproducible measurement for quantification of exercise calf perfusion and does not require signal normalization under these experimental conditions. Perfusion reserve measurements are less reliable, likely due to the inherently lower values for arterial input and tissue function at rest and resultant variability.

